# The E6 and E7 proteins of beta3 human papillomavirus 49 can deregulate both cellular and extracellular vesicles-carried microRNAs

**DOI:** 10.1186/s13027-022-00445-z

**Published:** 2022-06-15

**Authors:** Maria Vincenza Chiantore, Marco Iuliano, Roberta Maria Mongiovì, Sankhadeep Dutta, Massimo Tommasino, Paola Di Bonito, Luisa Accardi, Giorgio Mangino, Giovanna Romeo

**Affiliations:** 1 Department of Infectious Diseases, Istituto Superiore di Sanità, Rome, Italy; 2 Department of Medico-Surgical Sciences and Biotechnologies, Sapienza University of Rome - Polo Pontino, Latina, Italy; 3 Department of Oncogene Regulation, Chittaranjan National Cancer Institute, Kolkata, India; 4 Infections and Cancer Biology Group, International Agency for Research on Cancer, Lyon, France; 5 Present Address: Department of Pharmacy-Pharmaceutical Sciences, University of Bari A. Moro, Bari, Italy

**Keywords:** Human papillomavirus, microRNAs, Extracellular vesicles, Oncoproteins, HPV cancer

## Abstract

**Background:**

The β3 human papillomavirus (HPV)49 induces immortalization of primary keratinocytes through the action of E6 and E7 oncoproteins with an efficiency similar to alpha high risk (HR)-HPV16. Since HR-HPV oncoproteins are known to alter microRNA (miRNA) expression and extracellular vesicle (EV) production, we investigated the impact of HPV49 E6 and E7 proteins on miRNA profile and EV expression, and their involvement in the control of cell proliferation.

**Methods:**

The miRNA expression was evaluated by a miRNA array and validated by RT-qPCR in primary human keratinocytes immortalized by β3 HPV49 (K49) or α9 HR-HPV16 (K16), and in EVs from K49 and K16. The modulation of miRNA target proteins was investigated by immunoblotting analyses.

**Results:**

By comparing miRNA expression in K49 and K16 and the derived EVs, six miRNAs involved in HPV tumorigenesis were selected and validated. MiR-19a and -99a were found to be upregulated and miR-34a downregulated in both cell lines; miR-17 and -590-5p were upregulated in K49 and downmodulated in K16; miR-21 was downregulated only in K16. As for EV-carried miRNAs, the expression of miR-17, -19a, -21 and -99a was decreased and miR-34a was increased in K49 EVs. In K16 EVs, we revealed the same modulation of miR-19a, -34a, and -99a observed in producing cells, while miR-21 was upregulated. Cyclin D1, a common target of the selected miRNAs, was downmodulated in both cell lines, whereas cyclin-dependent kinase 4 was down-modulated in K49 but upregulated in K16.

**Conclusion:**

These data suggest that E6 and E7 proteins of β3 HPV49 and α9 HR-HPV16 affect key factors of cell cycle control by indirect mechanisms based on miRNA modulation.

**Supplementary Information:**

The online version contains supplementary material available at 10.1186/s13027-022-00445-z.

## Background

The Papillomaviridae family includes more than 400 human papillomaviruses (HPVs) classified into 5 genera (α, β, γ, μ, and ν) and characterized by the ability to infect cutaneous or mucosal epithelia [1]. Twelve mucosal α-HPV genotypes defined as high-risk (HR) are the causative agent of virtually all Squamous Cell Carcinomas (SCC) of the cervix, a high percentage of those in the ano-genital area and an increasing fraction of the head and neck cancers (HNSCC) [2, 3].

While the activities of oncogenic HPVs belonging to the α genus are well characterized, other HPVs associated to benign or asymptomatic infections may retain as yet unknown properties [4]. Many findings indicate that cutaneous β-HPVs are involved in skin carcinogenesis [5]. In animal models, the HPV38 and HPV49 members of the β genus can induce cancer in cooperation with UV radiation or chemical insult [6, 7].

HPV genome is composed of early (E) and late (L) genes that cumulatively regulate the virus life cycle. E6 and E7 genes code for two proteins able to interfere with several pathways of the host cell by targeting cell proteins, in particular the p53 and pRb tumor suppressors [8]. HR-HPV E6 and E7 oncoproteins hamper the activity of p53 and pRb respectively, through various mechanisms, the main one being degradation [9, 10].

Beta-Papillomavirus HPVs show cutaneous tropism, but the β3 species, including 4 genotypes (HPV49, 75, 76, and 115), was also detected into the oral mucosal epithelium [11]. Emerging evidence indicates that β3 human papillomavirus type 49 (HPV49) shares biological properties with mucosal HR-HPV type 16 (HPV16). Among the host processes deregulated by HPV16 E6 and E7 there is the expression of miRNAs. Interestingly, it was demonstrated that, when transduced into human primary keratinocytes, HPV49 E6 and E7 induce cell immortalization with a mechanism similar to HR-HPVs. In particular, HPV49 E6 promotes p53 degradation in an ubiquitin ligase enzyme E6AP-dependent manner [11, 12].

MicroRNAs (miRNAs) are small noncoding RNAs that post-transcriptionally regulate gene expression by negatively affecting the persistence of targeted mRNAs in the cell. MiRNAs are associated with almost all physiological and pathological cellular processes, included cancer. They can be divided into oncogenic or oncosuppressor miRNAs based on their respective ability to favor or counteract carcinogenesis [13, 14]. Oncogenic viruses such as HR-HPVs have been reported to alter host miRNA expression to induce carcinogenesis [15, 16].

Extracellular vesicles (EVs) are a heterogeneous family of membrane-limited nanoparticles released by cells. They are classified into three subtypes (exosomes, microvesicles and apoptotic bodies) based on their biogenesis and size, and can deliver DNA, mRNA, miRNA, proteins and lipids to recipient cells [17]. EVs can concur in several physiological and pathological cellular processes by altering the tumor microenvironment (TME) development and inducing the establishment of metastases [17–20]. In particular, miRNAs are loaded into EVs, thus playing an important role in intercellular communication, cancer cell proliferation and TME definition [21, 22]. Oncogenic viruses, including HR-HPVs [23], alter the EV cargo and release from infected cells in order to favor the infection and/or promote carcinogenesis [24, 25]. It has been shown that EVs contribute to the reprogramming of TME by delivering miRNAs to surrounding cells [25, 26]. In addition, it has been demonstrated that miRNAs can be loaded into exosomes through specific pathways [27]. Furthermore, E6 and E7 oncoproteins were shown to affect the miRNA cargo composition of EVs isolated from HPV positive cell lines [23].

Here we investigated whether the HPV49 genotype could support cell transformation by miRNA deregulation. We reported the expression analysis of miRNAs derived from primary human foreskin keratinocytes transduced with the E6 and E7 oncoproteins of either β3 HPV49 (K49) or α9 HR-HPV16 (K16), and from the corresponding EVs. The results showed an altered miRNA expression pattern in K49 and K16. Six miRNAs (miR-17, -19a, -21, -34a, -99a and -590-5p) deregulated in K49, K16 and related EVs were identified and selected for their relevance in HPV-associated carcinogenesis. The modulation of the expression of the selected miRNAs in cellular and vesicular extracts of K49 and K16 correlates with the alteration of CCND1 and CDK4, two cell cycle control factors with a pivotal role in HPV-induced carcinogenesis.

## Methods

### Cell cultures and treatments

Primary human foreskin keratinocytes were transduced with pLXSN49E6E7, pLXSN16E6E7 or pLXSN alone, as previously described by Caldeira et al. [9], and will be referred to as K49, K16 and HFK, respectively. HFK or K16 and K49 silenced for E6 and E7 by specific small interfering RNAs (siRNAs) (K16 sil. and K49 sil.) were used as controls. All cell types were cultured in KGM-Gold medium complemented with KGM-Gold Single Quots (Lonza, Basel, Switzerland) in a humidified atmosphere and 5.0% CO_2_ at 37 °C.

### HPV16 E6/E7 and HPV49 E6/E7 silencing

SiRNAs able to target HPV16 E6, HPV16 E7, HPV49 E6, HPV49 E7 mRNAs were designed and synthesized by Qiagen (Hilden, Germnay) as follows: HPV-16 E6 siRNAs sense 5'-GAGGUAUAUGACUUUGCUU-3', HPV-16 E6 siRNAs antisense 5'-AAGCAAAGUCAUAUACCUC-3'; HPV-16 E7 siRNAs sense 5'-AGGAGGAUGAAAUAGAUGG-3', HPV-16 E7 siRNAs sense antisense 5'-CCAUCUAUUUCAUCCUCCU-3'; HPV-49 E6 siRNAs sense 5'-GCATATCACGAGTTTACTAAT-3', HPV-49 E6 siRNAs antisense 5′-ATTAGTAAACTGCTGATATGC-3'; HPV-49 E7 siRNAs sense 5'-GCCACTGACGCTGCTATTAGA-3', HPV-49 E7 siRNAs antisense 5'-TCTAATAGCAGCGTCAGTGGC-3'. Silencing was performed by transfection following manufacturer’s instructions. Briefly, for a 100 mm diameter Petri dish, 3 μl of siRNA E6 (20 µM), 3 μl of siRNA E7 (20 µM) and 35 μl of HiPerfect Transfection Reagent (Qiagen, Hilden, Germany) were diluted in 500 μl of KGM-Gold medium (Lonza, Basel, Switzerland) without supplements and mixed by vortexing. After 10 min at room temperature, the transfection solution was added drop-wise onto the cells. The lower expression of HPV49 E6 or E7 and HPV16 E6 or E7 was confirmed by Real Time RT-PCR 96 h following the siRNA silencing.

### MiRNA extraction and TaqMan array human MicroRNA A card analysis

Cells were lysed by using mirVana miRNA detection kit (Applied Biosystems, Waltham, MA, USA) that allows detecting small RNAs, following the manufacturer’s procedure. The extracted RNA was retro-transcribed by TaqMan Micro-RNA Reverse Transcription Kit and Megaplex RT Primers (Applied Biosystems, Waltham, MA, USA). TaqMan Array Human MicroRNA A Card (Applied Biosystems, Waltham, MA, USA) was used to analyze the expression of 384 miRNA sequences in K49 and K16 cells compared to HFK. Data obtained were analyzed by using qPCR on Thermo Fisher Cloud (Thermo Fisher Scientific, Waltham, MA, USA) and setting global normalization, 35 as maximum Threshold Cycle (Ct) and HFK or K16 sil. as a reference.

### Extracellular vesicles (EVs) purification

K16 and K49 were seeded at 1 × 10^6^ cells in 100‐mm‐diameter Petri dishes (3 plates for each condition) and, 2 h later, silenced for E6 and E7 as previously described. After 96 h, supernatants were collected and EVs isolated by serial centrifugations. Supernatants were centrifuged at 500 × g for 10 min and 2000 × g for 10 min to remove detached cells and cellular debris, and then ultracentrifuged at 100,000 × g for 60 min. Pellets containing EVs were lysed to extract total RNA as described below.

### Real‑time RT‑PCR

Cells were seeded as described in the above section. After 96 h, after the collection of supernatants for EVs purification, cells were detached with trypsin EDTA 0.25% (Corning, Corning, NY, USA), washed twice in PBS, and an aliquot was lysed for total RNA extraction using the Total RNA Purification Kit (Norgen Biotech Corp., Thorold, ON, Canada). The extracted RNA was retro-transcribed by using the TaqMan Micro-RNA Reverse Transcription Kit while specific miRNA amplifications were performed by using TaqMan-based Small RNA assays following manufacturer’s instructions (Applied Biosystems, Waltham, MA, USA). For the determination of the rate of E6 and E7 silencing, total RNA was extracted from K16, K49 and the corresponding silenced cells using the Total RNA Purification Kit (Norgen Biotek Corp., Ontario, Canada). One μg of total RNA was retro‐transcribed using the Tetro cDNA synthesis kit (Meridian BioScience, Cincinnati, Ohio, USA), and cDNA products were analyzed by Real Time RT-PCR using the SensiMix SYBR Hi‐ROX Kit (Meridian Bioscience, Cincinnati, OH, USA). Data were normalized using HPRT‐1 as endogenous controls and expressed using the 2^−ΔΔCT^ method [28]. The gene-specific primers used to detect E6 and E7 of HPV16 and HPRT-1 mRNAs were selected from Chiantore et al. [16] while those used to detect E6 and E7 of HPV49 are the following: E6 FWD 5'-TGGTTGTTGTGCAGCTTGTG-3'; REV 5'-ATATTAGCCGCTGCTCGTCC-3'; E7 FWD 5'-TGCGAGTCTTCGTGTTAGCC-3’; E7 REV 5'-CACGACACTGAGGACACAAGA-3’.

### Western blot analysis

To analyze protein expression, K16, K16 sil., K49 and K49 sil. were lysed in UBI buffer (50 mM Tris pH 7.4, 1 mM EDTA, 0.1% NP40, 250 mM NaCl, 5 mM NaF, 0.5 mM NaVO3, pepstatin, PMSF, aprotinin, benzamidine, leupeptin). Total cell extracts were cleared by centrifugation and boiled in the presence of 5% 4–2-Mercaptoethanol and 0.01% bromophenol blue. Forty μg of total proteins were resolved on SDS-PAGE and transferred onto PVDF membrane (Amersham, Amersham, UK). Membranes were blocked with 5% dried skim milk dissolved in PBS-Tween 20 and incubated with the following primary antibodies: mouse anti-CCND1 (Sigma Aldrich, St. Louis, MO, USA), goat anti-CDK4 (Santa Cruz Biotechnology, Dallas, TX, USA); mouse anti-p53 (Santa Cruz Biotechnology); mouse anti-Actin (Santa Cruz Biotechnology, Dallas, TX, USA) and rabbit monoclonal anti-GAPDH (14C10, Cell Signaling, Danvers, MA, USA) as loading control. Immune complexes were detected with horseradish peroxidase-conjugated goat anti-rabbit, rabbit anti-mouse (Calbiochem, San Diego, CA, USA) and donkey anti-goat antiserums (Santa Cruz Biotechnology) followed by enhanced chemiluminescence reaction (Clarity Western ECL Substrate, Bio‐Rad).

### Principal component analysis (PCA)

**Fig. 1:**
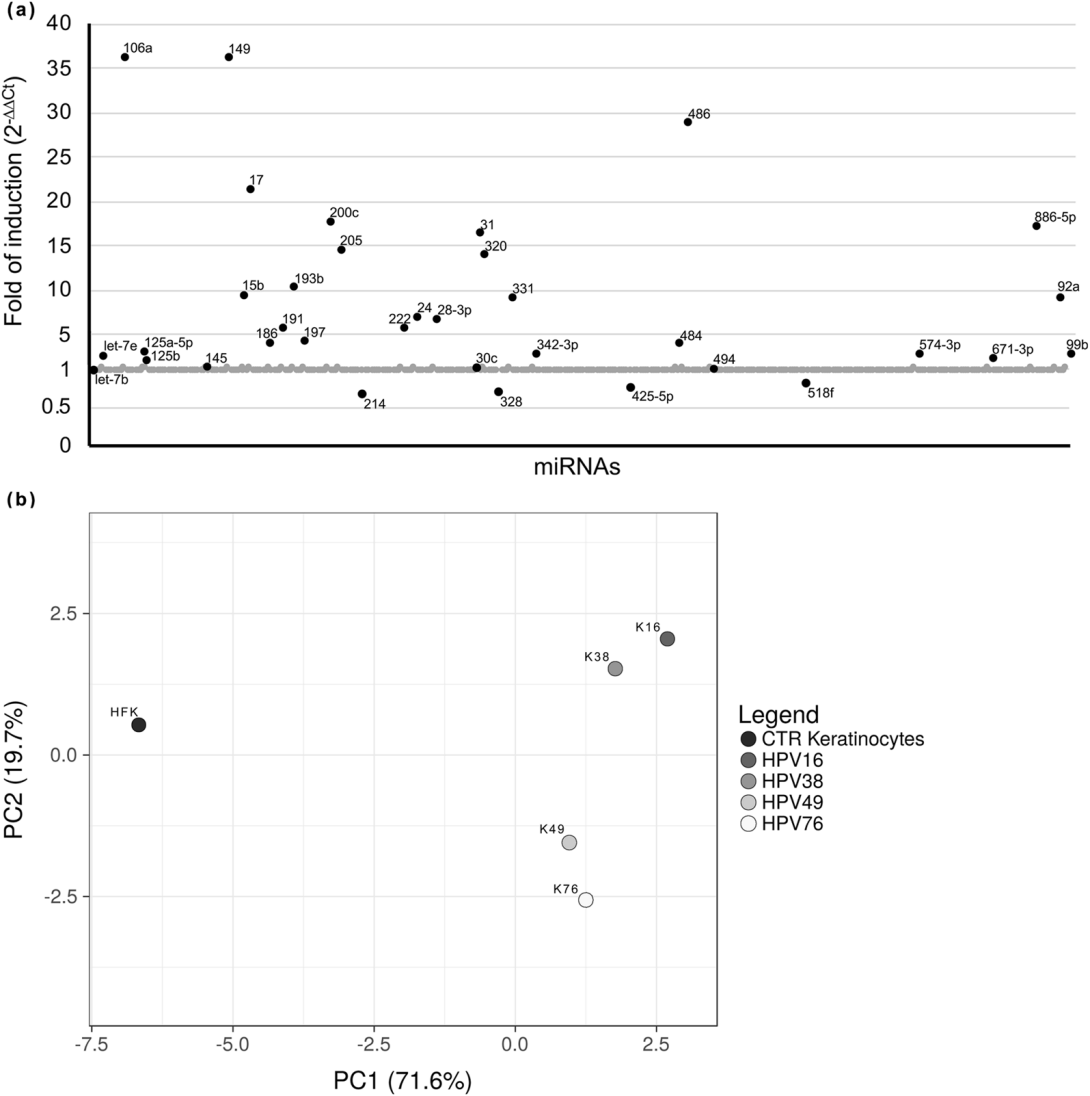
**a** MiRNA profiling in K49 compared to HFK cells. MiRNA analysis was performed using the TaqMan Array Human MicroRNA A Card. Results were expressed as fold of induction using the 2^−ΔΔCT^ method [28] and HFK as a reference. According to the 2^−ΔΔCT^ method, the value of non-deregulated MiRNAs was set to 1. Deregulated and selected miRNAs are indicated with black dots; non-deregulated miRNAs are plotted with gray dots. **b** Principal Component Analysis performed on ΔCts derived from the analysis of K16, K38, K49, K76 and HFK used as controls. Each dot represents the variance of 35 miRNAs, selected from the previous analysis on K49 cells, for each reference cell population (K16, K38, K49, K76 andHFK). PCA was performed by using ClustVis (https://biit.cs.ut.ee/clustvis/) algorithm

**Fig. 2:**
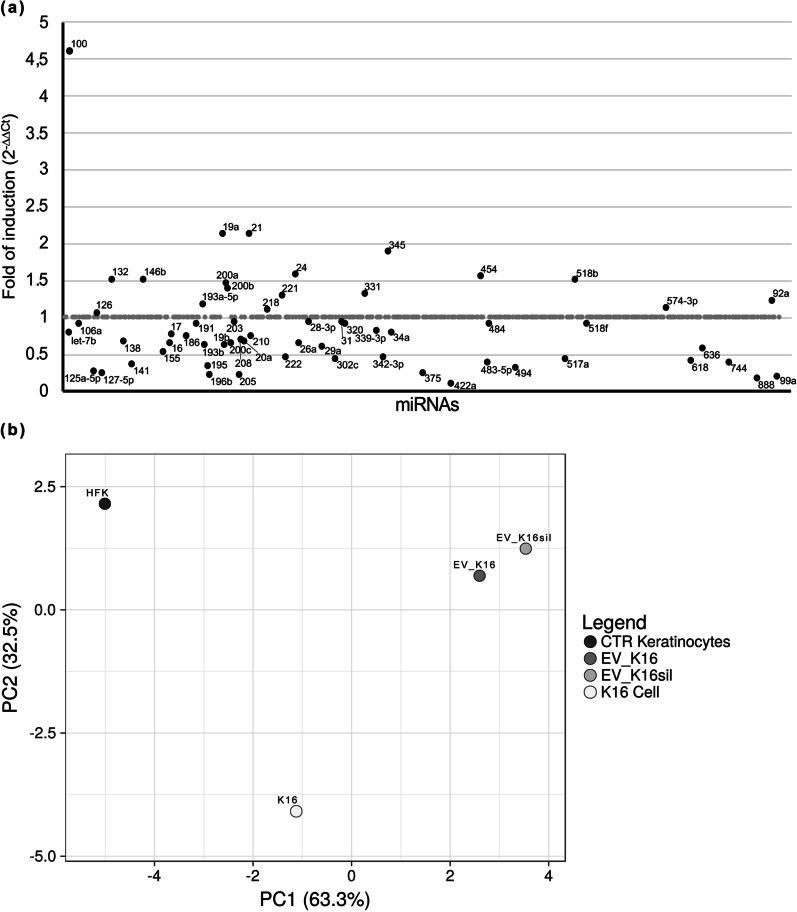
**a** MiRNA profiling on EV_K16 compared to EV_K16sil. Results were expressed as fold of induction or decrease using the 2^−ΔΔCT^ method, and EV_K16 sil., as a reference. According to the 2^−ΔΔCT^ method, the value of non-deregulated miRNAs was set to 1. The miRNAs not showing variation in the expression between EV_K16 and EV_K16 sil. are positioned on the value equal to one. The selected and deregulated miRNAs are indicated with black dots; non-deregulated miRNAs are plotted with gray dots. **b** Principal Component Analysis conducted on ΔCts derived from the analysis of EV_K16, EV_K16 sil., K16, keratinocytes and monocytes used as controls. Each dot represents the variance of 62 miRNAs selected from the previous analysis on EV_K16, for each reference cell population (EV_K16, EV_K16 sil., K16 and HFK). PCA was performed by using ClustVis (https://biit.cs.ut.ee/clustvis/) algorithm

PCA was performed by using ClustVis website by using the “PCA method” of the R package (https://biit.cs.ut.ee/clustvis/#editions; for more information see here [29]). Shortly, the analysis was conducted on ΔCt of all the miRNAs that were eligible for selection (black dots of Figs. 1a and 2a) from the TaqMan Array Human MicroRNA A Cards analysis of HFK, K49, EV K16 and EV K16 sil. To allow for a comparison, ΔCt derived from analysis performed on miRNAs derived from K16 and K38, previously published [16], and from HFK transduced with pLXSN76E6E7 (K76) were used. A list of ΔCt derived from a monocyte cell line was used as a control to verify the effectiveness of PCA (data not shown).

### MiRNA targets prediction

To determine a set of predicted targets for a selected miRNA DIANA Tools TarBase v.8 software (http://www.microrna.gr/tarbase) was used. The predicted targets, shown in Fig. S4b, were classified by using database management software (Microsoft Office Access, Redmond, WA, USA) to collect all the proteins sharing at least 3 of all the selected miRNAs as modulators.

### Statistical analysis

Two‐tail *P* value Mann‐Whitney t test was performed to compare two sample groups. *: *P* < 0.05; **: *P* < 0.01; ***: *P* < 0.001. All data are presented as mean ± SD.

## Results

### MicroRNAs expression is deregulated in K49 compared to HFK cells

The expression analysis of the most valuable and significant human miRNAs was performed in K49 by using TaqMan Array Human MicroRNA A Card as described in Materials and Methods.

The dispersion plot in Fig. 1a shows that 35 miRNAs were deregulated in K49 compared to HFK, used as a control. The complete list of deregulated miRNAs can be found on Additional file 1: Table 1S. HPV49 E6 and E7 can alter the expression of a wide range of miRNAs, as observed also for HPV16 and 38 E6/E7-transformed keratinocytes [16]. To establish whether HPV49 E6 and E7 deregulated miRNAs expression more like mucosal HR-HPV16 or cutaneous HPVs, a principal component analysis (PCA) was applied to the dataset (Fig. 1b). This analysis was performed using ClustVis website on ΔCt of all the miRNAs that were eligible for selection. The miRNA dataset for each cell type analyzed and its variability was reduced and represented as a single dot on a first principal component (PC)1 vs PC2 scatterplot.

The first component describes the majority of the variance and shows a great similarity among the dots representing HPV E6/E7-positive cells and HFK. Only by analyzing the second component, the differences among K49, K16 and K38 emerged. Dot K76 represents the miRNA dataset from keratinocytes transduced with E6 and E7 of cutaneous β3 HPV76 genotype and confirmed characterization of β3 species along PC2. Overall, PCA suggests that few miRNAs can discriminate cells transduced with E6/E7 of β3 HPVs from others.

### HPV-16 E6 and E7 expression can regulate the EV content of specific microRNAs

To define the possible role of E6 and E7 in the loading and spreading of miRNAs by EVs, a TaqMan-based miRNA A array was used to analyze hundreds of miRNAs associated to cancer in EVs derived from K16 cells (EV_K16) versus EVs derived from E6/E7 silenced K16 (EV_K16 sil.). The complete list of deregulated miRNAs can be found on Additional file 1: Table 2S. Sixty-two miRNAs were identified based on their deregulation level (Fig. 2a). Since E6 and E7 oncoproteins can deregulate the expression of several miRNAs detected in EVs, we wondered whether this deregulation could show a valuable difference among dataset of miRNAs from EVs derived from silenced or non-silenced K16 cells and from the producing K16 cells. To verify this hypothesis, we performed PCA also on miRNA datasets from EV_K16, EV_K16 sil., K16 and HFK (Fig. 2b). A minimal variation on PC1 among K16, EV_K16 and EV_K16 sil. was registered, displaying a similar miRNA profile between producing cells and derived EVs. The distance on PC2 axis from EV_K16, EV_K16 sil. and K16 indicates the possibility to find a small group of miRNAs that characterize the vesicular miRNA dataset compared to the cellular one. This difference seems not to be strictly dependent on the expression of E6 and E7. The dots representing the HFKs confirm the effectiveness and centrality of PC1 in discriminating the variability among different datasets, as previously observed (Fig. 1b).

### K49 and K16 are able to deregulate miR-17, -19a, -21, -34a, -99a, -590-5p both in cellular and in vesicular extracts

**Fig. 3:**
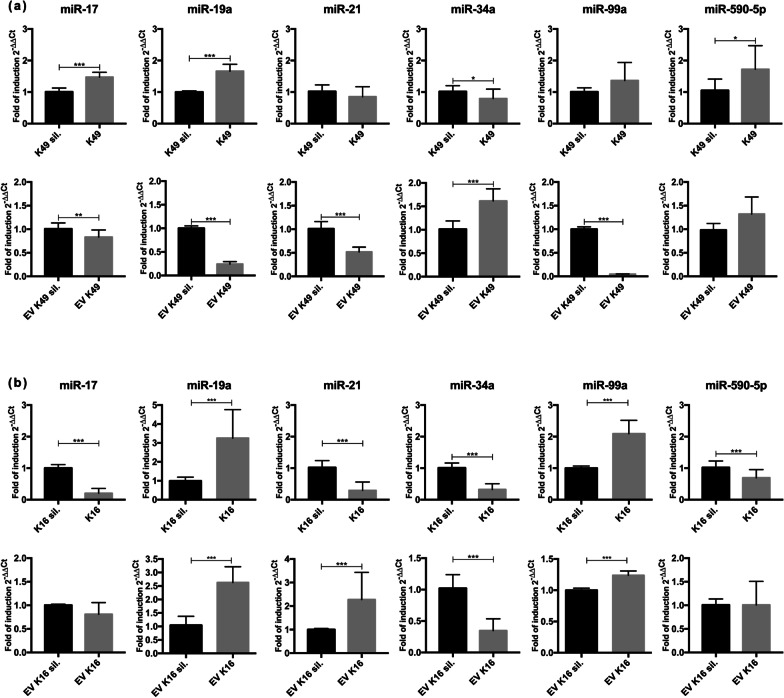
MiRNA expression analysis performed in **a** K49, K49 sil., EV_K49, EV_K49 sil. and **b** K16, K16 sil., EV_K16, EV_K16 sil.. Total RNA from each sample was purified as described in Materials and Methods. RNU6 was used as a calibrator and K49 sil., EV_K49 sil., K16 sil. and EV_K16 sil. were used as controls. Results were expressed as fold of induction or decrease using the 2^−ΔΔCT^ method [28]. The reported values represent the mean of three independent experiments ± the standard deviation. *P*-values (*P*) are indicated with asterisks. **P ≤ 0.01, *P ≤ 0.05

The analysis of the involvement of HPV E6 and E7 in miRNA deregulation was focused on selected miRNAs, whose expression was analyzed by Real Time RT-PCR both in cellular and vesicular RNA extracts from K49, K49 sil., K16 and K16 sil. (Fig. 3a, b). The selected miRNAs showed evident deregulation in the card array in both K49 and K16 cells. Moreover, based on the PCA results, we extended the choice to some miRNAs detected into K16 EVs with the hypothesis that they might be important also in the EVs derived from K49 cells. MiR-17, -19a, -21, -34a, -99a and -590-5p were analyzed in K49 and K16 cells, and EVs compared to those from cells silenced for E6 and E7 used as a control. Quantitative RT-PCR results in K49 cellular extracts showed that miR-17, -19a and -590-5p were significantly upregulated, and that miR-34a was downmodulated (Fig. 3a). Conversely, miR-17, -19a, -21 and -99a expression was significantly decreased and miR-34a expression was increased in K49 EVs. The analysis of these miRNAs in K16 cells and EVs (Fig. 3b) showed the downmodulation of miR-17, -21, -34a and miR-590-5p, and the upregulation of miR-19a and -99a in cell extracts. The same results were obtained in K16 EVs, except for miR-21.

### E6 and E7 oncoproteins of HPV49 and HPV16 affect CCND1, CDK4 and p53 expression

**Fig. 4:**
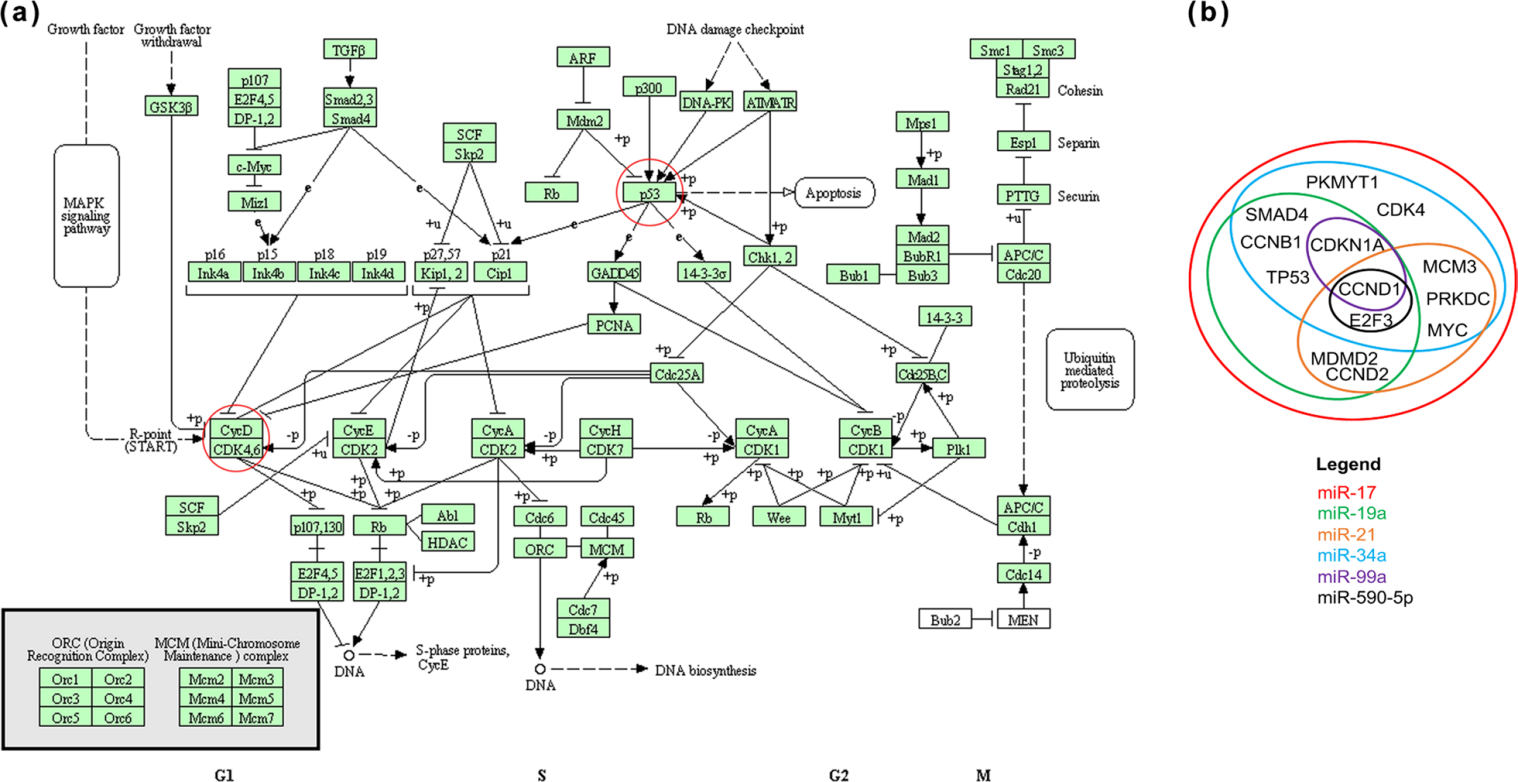
**a** Cell cycle pathways downloaded from http://www.microrna.gr/tarbase. **b** Venn diagram with predicted targets sharing at least 3 of the selected miRNAs as modulators and linked to cell cycle pathways selected by DIANA Tools TarBase v.8 software (http://www.microrna.gr/tarbase) and classified by database management software (Microsoft Office Access, Redmond, WA, USA)

After the selection of the miRNAs (miR-17, -19a, -21, -34a, -99a and -590-5p) deregulated by HPV49 and HPV16, we proceeded to identify common protein targets of these miRNAs by using the DIANA Tools software TarBase v.8 (Fig. 4b). Protein targets were identified among proteins belonging to the cell cycle regulating pathways important for HPV-associated carcinogenesis, as shown in Fig. 4a.

**Fig. 5:**
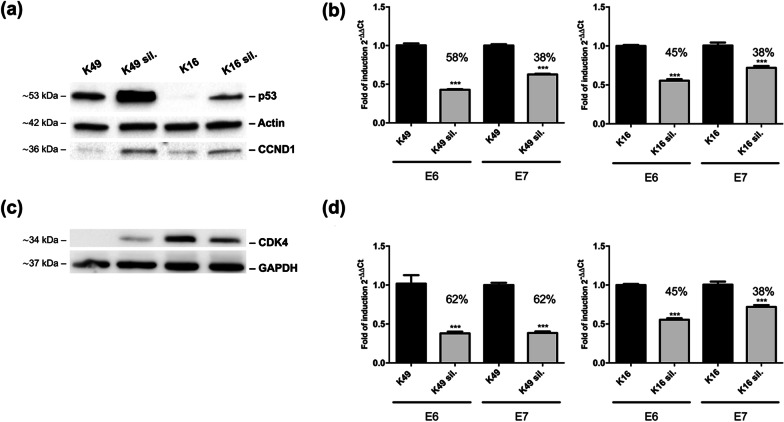
Western blot analysis of CCND1, p53 (**a**), and CDK4 (**c**) in K49 and K16 or K49 and K16 silenced for E6 and E7. Whole cell extracts were resolved on SDS-PAGE and transferred onto PVDF membrane. Immunoblotting was performed as reported in Materials and methods. Actin (**a**) and GAPDH (**c**) were used as an internal loading control. Two representative experiments out of five performed are shown. Rate of E6 and E7 mRNA downmodulation following siRNA silencing are shown for each experiment in (**b**) and (**d**)

MiR-17, -19a, -21, -34a, -99a e -590-5p showed a similar set of targets. Interestingly, cyclin D1 (CCDN1) is a common target for all miRNAs analyzed. It belongs to the family of cyclin-dependent kinases (CDK) which are fundamental in the regulation of cell cycle. CDK4 and p53 are common targets to three of the analyzed microRNAs. All these proteins are pivotal in the development of HPV-associated tumorigenesis. The levels of CCND1, CDK4 and p53 targets were investigated by western blot analysis in K49 and K16 compared to K49 and K16 silenced for E6/E7 cells. Figure 5a shows that CCND1 expression was increased in K49 sil. and K16 sil. with respect to the non-silenced cell lines. The same result was observed for p53, although for this oncosuppressor it must be taken into account that the silencing of E6 itself leads to the rescue of p53 levels. CDK4 expression was upregulated in K49 sil. while it was downregulated in K16 sil. (Fig. 5c). The silencing rates in K49 and K16 cells relative to western blot analyses are shown in Fig. 5b, d.

The modulation of some targets of the miRNAs deregulated by E6 and E7 indicates that the HPV oncoproteins can affect, with different mechanisms, specific factors with key roles in cell cycle progression.

## Discussion

It is known that the oncogenic potential of HPV lies in the transforming activity of the E6 and E7 viral proteins, which, interacting with different host cell proteins, can interfere with the main cellular processes such as the cell cycle, apoptosis and shortening of telomeres [30]. Twelve high-risk mucosal papillomaviruses belonging to the Alpha genus, including HPV16, are known to be the cause of squamous carcinoma of the uterine cervix (SCC) and of other tumors. On the other hand, the role of cutaneous β-HPVs genus in the carcinogenesis of human skin keratinocytes is not yet fully understood. β-HPVs are abundantly present in healthy skin as well as in skin tumors such as actinic keratosis and keratoacanthoma, indicating that some HPV genotypes can be involved in the development of cutaneous carcinomas in cooperation with UV radiation [6, 31, 32]. Recent studies found that β-HPVs, and in particular β3, which includes HPV49, HPV75, HPV76 and HPV115, can also infect other anatomical areas besides the skin, such as the epithelium of the oral mucosa, eyebrow hair and external genitalia [12]. Interestingly, the β3 HPV49 shares several biological properties with the mucosal HR α-HPV16 [12].

Cutaneous β-HPVs seem to act in the early stage of carcinogenesis by targeting cellular proteins such as pRb and p53, but only a limited number of studies have investigated the transformation capacity of β-HPVs in primary keratinocytes [6, 16]. In the context of natural infection, β-HPVs have developed strategies to keep infected cells in a proliferative state in order to effectively complete their life cycle in the skin [33].

HPV49 E6 oncoprotein, similar to E6 of mucosal HPV16, binds to p53 promoting its degradation through the proteasome pathway; however, the possibility has been highlighted that the β3 HPV E6 and E7 proteins may use alternative and not yet characterized mechanisms to block p53 function. In addition, E6 and E7 of HPV16 and HPV49 are capable of immortalizing primary human keratinocytes with similar efficiency levels [12].

It has also been reported that the action of the HPV E6 and E7 viral oncoproteins can be carried out at the post-transcriptional level through the modulation of microRNAs [14, 16, 34, 35].

The present work investigated whether cutaneous β3 HPV49 is able to deregulate selected miRNAs involved in tumorigenesis promotion as already observed for mucosal α-HPV16 and cutaneous β2 HPV38 [16, 34]. MiRNA profiling of keratinocytes transduced with E6 and E7 from cutaneous β3 HPV49 (K49) showed that the expression of HPV49 E6 and E7 leads to the modulation of different miRNAs compared to primary keratinocytes transduced with the pLXSN plasmid (HFK), used as a control. In particular, 35 miRNAs whose expression was significantly regulated, were identified (Fig. 1a). Principal Component Analysis (Fig. 1b) was performed to investigate whether the patterns of miRNA expression altered by HPV49 E6 and E7 were similar to those already reported for mucosal α-HPV16 and cutaneous β2 HPV38 miRNAs [16]. PCA suggests that few miRNAs could discriminate cells transduced with E6 and E7 of β3 HPVs from the others.

The importance of EVs in the HPV-associated tumorigenesis process is increasingly evident [23, 24, 36]. It has been shown that in tumor cells EVs release is altered and implements a functional modification of the cellular microenvironment thereby reducing the antitumor immune response. In addition, microRNAs can play an important role in HPV E6/E7-induced transformation not only as free molecules but also as EV cargo components [16, 37, 38]. EVs derived from K16 cells (EV_K16) were isolated to analyze miRNA expression by TaqMan-based miRNA array in order to investigate the presence of specific miRNAs potentially involved in HPV-induced tumorigenesis.

In this case, we used E6/E7 silenced K16 cells (EV_K16 sil.) as controls to overcome the low EV release observed in HFK and the tendency of these untransformed cells to go senescence (data not shown). Through this approach, 62 deregulated miRNAs were identified (Fig. 2a). PCA analysis showed a minimal variation on PC1 among K16, EV_K16 and EV_K16 sil., suggesting the existence of a similar expression pattern between the cells and their respective EVs (Fig. 2b). Indeed, it is known that exosomes can use specific mechanism for miRNA uploading [27], thereby, it might be reasonable to find a superimposable miRNA distribution in the released EVs and the parental cells. Only by analyzing the distance on PC2 from EV_K16, EV_K16 sil. and K16 it is possible to find a small group of miRNAs able to discriminate between vesicular and cellular miRNA patterns. In this work, the analysis on the six selected miR-17, -19a, -21, -34a, -99a and -590-5p was performed based on the card array results of K49, EV_K16 and EV_K16 sil., and on literature data [16, 39–41]. Deregulation of these miRNAs was confirmed by Real Time RT-PCR both in cellular lysates and vesicular RNA extracts from K49 and K16 compared to the same cell lines silenced for E6 and E7, used as controls.

In K49 cells, miR-17, -19a and -590-5p were significantly upregulated and miR-34a was downmodulated. Conversely, miR-17, -19a, -21 and -99a expression was decreased and miR-34a expression increased in K49 EVs (Fig. 3a).

In both K16 cells and EVs we observed the down-modulation of miR-17, -34a and -590-5p and the upregulation of miR-19a and -99a. Only miR-21 had different regulation in K16 cells and EVs, being downmodulated in cell lysates and upregulated in vesicular extracts (Fig. 3b).

MiR-17 and -19a belong to the miR-17–92 cluster, a highly conserved polycistronic miRNA cluster expressed in a wide range of tumors that however may have antitumor properties [42, 43]. In particular, miR-17 is generally considered an oncogene, since it is upregulated in many tumors including HPV-induced cervical cancer [37, 39, 44]. High levels of this miRNA have been found in the serum of patients with different types of cancer [45, 46]. Nevertheless, it has been reported that miR-17 also possesses metastasis suppressor functions [47]. MiR-21 is associated with oncogenesis in many different cancers, indicating that it is a potential clinical biomarker. Elevated levels of miR-21 have also been detected in cervical cancer [39]. Moreover, it has been suggested that miR-21-enriched exosomes may play a role in tumor cell proliferation, migration, and invasion [48]. It is known that miR-34a is a target of p53, since its promoter contains the p53 binding site. In turn, p53 is also a target of miR-34a. Indeed, the interplay between miR-34a and p53 is very complex: p53 induces miR-34a transcription, but miR-34a can regulate the expression and activity of p53 protein both positively and negatively [48, 49]. MiR-34a is downregulated in cervical cancer and in HPV16 positive cells [16, 39, 50, 51]. MiR-99 belongs to a miRNA family with important roles in cancer progression and immunity. Levels of members of miR-99 family were shown to be down-regulated in tumor cells [52]. MiR-590-5p was reported to be upregulated as a tumor oncogene in human cervical cancer and other types of cancers but it can also exert an anti-tumorigenic role, as in colorectal and breast tumors [53]. Therefore, it seems clear that all these miRNAs can act as both oncomiRs and tumor suppressor miRNAs, depending on the type of tumor and the target genes they affect [54]. The difference between the results of miRNA modulation by HPV49 and HPV16 E6 and E7 probably reflects both the cellular context (*i.e.,* keratinocytes expressing proteins from two different HPV genotypes) and the different regulation of miRNA targets.

The identification of a specific target for a given miRNA is a particularly complex process because each miRNA targets multiple potential mRNAs and, conversely, the same target is regulated by several miRNAs [55].

The amount of validated and predicted targets for each miRNA is very high, so we established a “rule of engagement” to make a choice. In particular, the choice was based on targets belonging to central pathways in the HPV-associated tumorigenic process as this could support the possibility of an effective role of miRNAs in the process. Although several viral-cellular pathways involved in the induction of HPV-related carcinoma have been identified, those well-characterized are involved in safeguarding the integrity of the cellular genome by inducing, in the presence of DNA damage, the cell cycle deregulation (Fig. 4a). From the analysis of the molecular pathways of our interest, it emerged that the only target gene common to all selected miRNAs (miR-17, -19a, -21, -34a, -99a and -590- 5p), was cyclin D1 (CCND1) (Fig. 4b).

Cyclin D1 is involved in the early stages of the cell cycle and acts as a regulatory subunit of CDK4 or CDK6 kinases, also encoded by target genes of the selected microRNAs [56]. The activity of CCDN1 is essential for the transition from the G1 phase to the S phase of the cell cycle [57].

In our study, we focused on CCDN1 and CDK4, as the cyclinD1-CDK4 dimer normally inhibits the pRb protein allowing the E2F transcription factor to transcribe the genes necessary for entry into the S phase, while in an HPV-mediated infection E7 binds to pRb thus modifying the normal control of the cell cycle [58, 59].

In addition to the CCND1 target gene, the TP53 target mRNA, common to miR-17, -19a and -34a, was also identified. Normally the keratinocytes, following DNA mutations due to various causes such as UV rays or exposure to cancerogenic substances, respond by activating the DNA damage repair pathways (Fig. 4a), and inducing the cell cycle arrest or, if the damage is not repairable, apoptosis. On the other hand, HPV-infected keratinocytes express E6 and E7 viral proteins able to inhibit these molecular mechanisms [6].

Western blot analysis performed in K49 and K16 compared to E6/E7-silenced cells demonstrated that CCND1 expression is upregulated in K49 sil. and K16 sil. whereas CDK4 expression is upregulated in K49 sil. but downregulated in K16 sil. (Fig. 5a, c). Regarding p53, we observed the upregulation both in K49 and K16, as expected since the silencing of E6 itself leads to the rescue of p53 levels. However, we cannot exclude that this upregulation is also the result of the modulation of the miRNAs targeting p53.

Taken together, these results indicate that HPV49 and HPV16 oncoproteins can affect the levels of cellular regulatory factors that are targets of miRNAs involved in the tumorigenesis process. Clearly, HPV E6 and E7 can exert their function on these and other regulatory factors by different direct and indirect mechanisms, including miRNA modulation. However, even if the interplay among HPVs, miRNAs and cellular regulators is very complex, the action of different HPV E6 and E7 oncoproteins with transformation properties converges towards the same pathways with a key role in cell cycle control [60]. Although our results on the expression of miRNAs and their targets in keratinocytes transduced by β3 HPV49 and α9 HPV16 are not always superimposable, K49 and K16 seem to affect the same cellular pathways to induce cell transformation and cancer.

## Conclusions

We identified miRNAs with a possible pivotal role in the complex tumorigenic mechanism associated with HPV infection. We showed that HPV belonging to different genotypes and with different tropism could converge in the way of acting. All this may shed new light on the plasticity of the virus-host adaptation and the possibility of interaction among different genotypes during persistent HPV infection. Elucidating the role of EVs and microRNAs in HPV carcinogenesis could contribute to the advancement of knowledge of the molecular mechanisms underlying the HPV-host interaction and to the definition of the role of different genotypes in different HPV-associated types of cancer.

It is therefore important to analyze the effects of the EV miRNAs on the expression of potential targets in the acceptor HPV-negative cells in order to broaden and deepen the study of the role of miRNAs in the HPV-induced tumorigenesis. The study of the complex mechanism underlying the HPV activity in the host cell could open up new scenarios regarding the deregulation of cellular processes during HPV-associated tumorigenesis, highlighting the fundamental role of small non-coding RNAs also in the tumor microenvironment.

## Supplementary Information

**Additional file 1: Table S1**. Data from miRNAs profiling on K49 compared to HFK cells. MiRNAs analysis conduced using the TaqMan® Array Human MicroRNA Card assay. By escluding control miRNAs (i.e., RNU6, RNU44, RNU48) and miRNAs refer to other species (i.e., ath, mmus) 360 miRNAs have been analyzed. The seleceted miRNAs (bolded) have been chosen based on their expression level (Cq < 31 at least in one of the samples) and fold of induction different from 1. **Table S2**. Data from miRNAs profiling on EV_K16 compared toEV_K16sil. MiRNAs analysis conduced using the TaqMan® Array Human MicroRNA Card assay. By escluding control miRNAs (i.e., RNU6, RNU44, RNU48) and miRNAs refer to other species (i.e., ath, mmus) 360 miRNAs have been analyzed. The seleceted miRNAs (bolded) have been chosen based on their expression level (Cq < 31 at least in one of the samples) and fold of induction different from 1.

## Data Availability

The datasets used and/or analysed during the current study are available from the corresponding author on reasonable request.

## Abbreviations

HPV: Human papilloma virus
HR: High risk
miRNAs: MicroRNAs
K49: β3 HPV49 immortalized primary human keratinocytes
K16: α9 HR-HPV16 immortalized primary human keratinocytes
EVs: Extracellular vesicles
TME: Tumor microenvironment
HFK: Primary human foreskin keratinocytes transduced with pLXSN
siRNAs: Small interfering RNAs
PCA: Principal component analysis
K76: HFK transduced with pLXSN76E6E7
CCDN1: Cyclin D1
EV_K16: EVs derived from K16 cells
EV_K16 sil.: EVs derived from E6/E7 silenced K16
EV_K49: EVs derived from K49 cells
EV_K49 sil.: EVs derived from E6/E7 silenced K49
CDK: Cyclin-dependent kinases
SCC: Squamous carcinoma of the uterine cervix

## References

[1] Van Doorslaer K, Li Z, Xirasagar S, Maes P, Kaminsky D, Liou D (2017). The papillomavirus episteme: a major update to the papillomavirus sequence database. Nucleic Acids Res.

[2] Castellsagué X, Alemany L, Quer M, Halec G, Quirós B, Tous S (2016). HPV involvement in head and neck cancers: comprehensive assessment of biomarkers in 3680 patients. J Natl Cancer Inst.

[3] de Martel C, Plummer M, Vignat J, Franceschi S (2017). Worldwide burden of cancer attributable to HPV by site, country and HPV type. Int J Cancer.

[4] Egawa N, Doorbar J (2017). The low-risk papillomaviruses. Virus Res.

[5] Tommasino M (2019). HPV and skin carcinogenesis. Papillomavirus Res.

[6] Tommasino M (2017). The biology of beta human papillomaviruses. Virus Res.

[7] Viarisio D, Robitaille A, Müller-Decker K, Flechtenmacher C, Gissmann L, Tommasino M (2019). Cancer susceptibility of beta HPV49 E6 and E7 transgenic mice to 4-nitroquinoline 1-oxide treatment correlates with mutational signatures of tobacco exposure. Virology.

[8] Hebner CM, Laimins LA (2006). Human papillomaviruses: basic mechanisms of pathogenesis and oncogenicity. Rev Med Virol.

[9] Caldeira S, Zehbe I, Accardi R, Malanchi I, Dong W, Giarrè M (2003). The E6 and E7 proteins of the cutaneous human papillomavirus type 38 display transforming properties. J Virol.

[10] Accardi R, Dong W, Smet A, Cui R, Hautefeuille A, Gabet AS (2006). Skin human papillomavirus type 38 alters p53 functions by accumulation of deltaNp73. EMBO Rep.

[11] Viarisio D, Müller-Decker K, Zanna P, Kloz U, Aengeneyndt B, Accardi R (2016). Novel ß-HPV49 transgenic mouse model of upper digestive tract cancer. Cancer Res.

[12] Minoni L, Romero-Medina MC, Venuti A, Sirand C, Robitaille A, Altamura G (2020). Transforming properties of beta-3 human papillomavirus E6 and E7 proteins. mSphere..

[13] Tito C, De Falco E, Rosa P, Iaiza A, Fazi F, Petrozza V (2021). Circulating microRNAs from the molecular mechanisms to clinical biomarkers: a focus on the clear cell renal cell carcinoma. Genes (Basel).

[14] Tornesello ML, Faraonio R, Buonaguro L, Annunziata C, Starita N, Cerasuolo A (2020). The role of microRNAs, long non-coding RNAs, and circular RNAs in cervical cancer. Front Oncol.

[15] Vojtechova Z, Tachezy R (2018). The role of miRNAs in virus-mediated oncogenesis. Int J Mol Sci.

[16] Chiantore MV, Mangino G, Iuliano M, Zangrillo MS, De Lillis I, Vaccari G (2016). Human papillomavirus E6 and E7 oncoproteins affect the expression of cancer-related microRNAs: additional evidence in HPV-induced tumorigenesis. J Cancer Res Clin Oncol.

[17] Minciacchi VR, Freeman MR, Di Vizio D (2015). Extracellular vesicles in cancer: exosomes, microvesicles and the emerging role of large oncosomes. Semin Cell Dev Biol.

[18] Clancy J, D'Souza-Schorey C (2018). Extracellular vesicles in cancer: purpose and promise. Cancer J.

[19] Chang WH, Cerione RA, Antonyak MA (2021). Extracellular vesicles and their roles in cancer progression. Methods Mol Biol.

[20] Becker A, Thakur BK, Weiss JM, Kim HS, Peinado H, Lyden D (2016). Extracellular vesicles in cancer: cell-to-cell mediators of metastasis. Cancer Cell.

[21] Hu W, Liu C, Bi ZY, Zhou Q, Zhang H, Li LL (2020). Comprehensive landscape of extracellular vesicle-derived RNAs in cancer initiation, progression, metastasis and cancer immunology. Mol Cancer.

[22] Jeffries J, Zhou W, Hsu AY, Deng Q (2019). miRNA-223 at the crossroads of inflammation and cancer. Cancer Lett.

[23] Honegger A, Leitz J, Bulkescher J, Hoppe-Seyler K, Hoppe-Seyler F (2013). Silencing of human papillomavirus (HPV) E6/E7 oncogene expression affects both the contents and the amounts of extracellular microvesicles released from HPV-positive cancer cells. Int J Cancer.

[24] Chiantore MV, Mangino G, Zangrillo MS, Iuliano M, Affabris E, Fiorucci G (2015). Role of the microenvironment in tumourigenesis: focus on virus-induced tumors. Curr Med Chem.

[25] Iuliano M, Mangino G, Chiantore MV, Di Bonito P, Rosa P, Affabris E (2021). Virus-induced tumorigenesis and IFN system. Biology (Basel)..

[26] Tan S, Xia L, Yi P, Han Y, Tang L, Pan Q (2020). Exosomal miRNAs in tumor microenvironment. J Exp Clin Cancer Res.

[27] Villarroya-Beltri C, Gutiérrez-Vázquez C, Sánchez-Cabo F, Pérez-Hernández D, Vázquez J, Martin-Cofreces N (2013). Sumoylated hnRNPA2B1 controls the sorting of miRNAs into exosomes through binding to specific motifs. Nat Commun.

[28] Livak KJ, Schmittgen TD (2001). Analysis of relative gene expression data using real-time quantitative PCR and the 2(-Delta Delta C(T)) Method. Methods.

[29] Metsalu T, Vilo J (2015). ClustVis: a web tool for visualizing clustering of multivariate data using principal component analysis and heatmap. Nucleic Acids Res.

[30] Pal A, Kundu R (2019). Human papillomavirus E6 and E7: the cervical cancer hallmarks and targets for therapy. Front Microbiol.

[31] Donà MG, Chiantore MV, Gheit T, Fiorucci G, Vescio MF, La Rosa G (2019). Comprehensive analysis of β- and γ-human papillomaviruses in actinic keratosis and apparently healthy skin of elderly patients. Br J Dermatol.

[32] Galati L, Brancaccio RN, Robitaille A, Cuenin C, Luzi F, Fiorucci G (2020). Detection of human papillomaviruses in paired healthy skin and actinic keratosis by next generation sequencing. Papillomavirus Res.

[33] Cornet I, Bouvard V, Campo MS, Thomas M, Banks L, Gissmann L (2012). Comparative analysis of transforming properties of E6 and E7 from different beta human papillomavirus types. J Virol.

[34] Chiantore MV, Mangino G, Iuliano M, Zangrillo MS, De Lillis I, Vaccari G (2017). IFN-β antiproliferative effect and miRNA regulation in human papilloma virus E6- and E7-transformed keratinocytes. Cytokine.

[35] Shen S, Zhang S, Liu P, Wang J, Du H (2020). Potential role of microRNAs in the treatment and diagnosis of cervical cancer. Cancer Genet.

[36] Yuan Y, Cai X, Shen F, Ma F (2021). HPV post-infection microenvironment and cervical cancer. Cancer Lett.

[37] Honegger A, Schilling D, Bastian S, Sponagel J, Kuryshev V, Sültmann H (2015). Dependence of intracellular and exosomal microRNAs on viral E6/E7 oncogene expression in HPV-positive tumor cells. PLoS Pathog.

[38] Chiantore MV, Mangino G, Iuliano M, Capriotti L, Di Bonito P, Fiorucci G (2020). Human papillomavirus and carcinogenesis: novel mechanisms of cell communication involving extracellular vesicles. Cytokine Growth Factor Rev.

[39] Zheng ZM, Wang X (2011). Regulation of cellular miRNA expression by human papillomaviruses. Biochim Biophys Acta.

[40] Santos JMO, Peixoto da Silva S, Costa NR, Gil da Costa RM, Medeiros R (2018). The role of MicroRNAs in the metastatic process of high-risk HPV-induced cancers. Cancers (Basel)..

[41] Mo W, Tong C, Zhang Y, Lu H (2015). microRNAs' differential regulations mediate the progress of human papillomavirus (HPV)-induced cervical intraepithelial neoplasia (CIN). BMC Syst Biol.

[42] Hossain A, Kuo MT, Saunders GF (2006). Mir-17-5p regulates breast cancer cell proliferation by inhibiting translation of AIB1 mRNA. Mol Cell Biol.

[43] Gong AY, Eischeid AN, Xiao J, Zhao J, Chen D, Wang ZY (2012). miR-17-5p targets the p300/CBP-associated factor and modulates androgen receptor transcriptional activity in cultured prostate cancer cells. BMC Cancer.

[44] Hasanzadeh M, Movahedi M, Rejali M, Maleki F, Moetamani-Ahmadi M, Seifi S (2019). The potential prognostic and therapeutic application of tissue and circulating microRNAs in cervical cancer. J Cell Physiol.

[45] Wang M, Gu H, Wang S, Qian H, Zhu W, Zhang L (2012). Circulating miR-17-5p and miR-20a: molecular markers for gastric cancer. Mol Med Rep.

[46] Zeng X, Xiang J, Wu M, Xiong W, Tang H, Deng M (2012). Circulating miR-17, miR-20a, miR-29c, and miR-223 combined as non-invasive biomarkers in nasopharyngeal carcinoma. PLoS ONE.

[47] Fan M, Sethuraman A, Brown M, Sun W, Pfeffer LM (2014). Systematic analysis of metastasis-associated genes identifies miR-17-5p as a metastatic suppressor of basal-like breast cancer. Breast Cancer Res Treat.

[48] Li B, Cao Y, Sun M, Feng H (2021). Expression, regulation, and function of exosome-derived miRNAs in cancer progression and therapy. FASEB J.

[49] Kalfert D, Ludvikova M, Pesta M, Ludvik J, Dostalova L, Kholová I (2020). Multifunctional roles of miR-34a in cancer: a review with the emphasis on head and neck squamous cell carcinoma and thyroid cancer with clinical implications. Diagnostics (Basel)..

[50] Liu M, Wang W, Chen H, Lu Y, Yuan D, Deng Y (2020). miR-9, miR-21, miR-27b, and miR-34a expression in HPV16/58/52-infected cervical cancer. Biomed Res Int.

[51] Li J, Yu L, Shen Z, Li Y, Chen B, Wei W (2016). miR-34a and its novel target, NLRC5, are associated with HPV16 persistence. Infect Genet Evol.

[52] Eniafe J, Jiang S (2021). MicroRNA-99 family in cancer and immunity. Wiley Interdiscip Rev RNA.

[53] Jia G, Tang Y, Deng G, Fang D, Xie J, Yan L (2019). miR-590-5p promotes liver cancer growth and chemotherapy resistance through directly targeting FOXO1. Am J Transl Res.

[54] Lee Y, El Andaloussi S, Wood MJ (2012). Exosomes and microvesicles: extracellular vesicles for genetic information transfer and gene therapy. Hum Mol Genet.

[55] Díaz-González SEM, Deas J, Benítez-Boijseauneau O, Gómez-Cerón C, Bermúdez-Morales VH, Rodríguez-Dorantes M (2015). Utility of microRNAs and siRNAs in cervical carcinogenesis. Biomed Res Int.

[56] Hamid NA, Brown C, Gaston K (2009). The regulation of cell proliferation by the papillomavirus early proteins. Cell Mol Life Sci.

[57] Bao Y, Gabrielpillai J, Dietrich J, Zarbl R, Strieth S, Schröck F (2021). Fibroblast growth factor (FGF), FGF receptor (FGFR), and cyclin D1 (CCND1) DNA methylation in head and neck squamous cell carcinomas is associated with transcriptional activity, gene amplification, human papillomavirus (HPV) status, and sensitivity to tyrosine kinase inhibitors. Clin Epigenetics.

[58] Fiorucci G, Chiantore MV, Mangino G, Percario ZA, Affabris E, Romeo G (2012). Cancer regulator microRNA: potential relevance in diagnosis, prognosis and treatment of cancer. Curr Med Chem.

[59] Johnson ME, Cantalupo PG, Pipas JM (2018). Identification of head and neck cancer subtypes based on human papillomavirus presence and E2F-regulated gene expression. mSphere.

[60] Moody CA, Laimins LA (2010). Human papillomavirus oncoproteins: pathways to transformation. Nat Rev Cancer.

